# False-Positive Galactomannan Test Results in Multiple Myeloma

**DOI:** 10.3390/diseases13040118

**Published:** 2025-04-17

**Authors:** Shingen Nakamura, Yusaku Maeda, Ryohei Sumitani, Masahiro Oura, Kimiko Sogabe, Hikaru Yagi, Shiro Fujii, Takeshi Harada, Ken-ichi Matsuoka, Hirokazu Miki

**Affiliations:** 1 Department of Community Medicine and Medical Science, Tokushima University Graduate School of Biomedical Sciences, Tokushima 7708503, Japan; 2 Department of Hematology, Endocrinology and Metabolism, Tokushima University Graduate School of Biomedical Sciences, Tokushima 7708503, Japan; 3 Division of Transfusion Medicine and Cell Therapy, Tokushima University Hospital, Tokushima 7708503, Japan

**Keywords:** galactomannan, aspergillus, multiple myeloma

## Abstract

Background/Objectives: Invasive pulmonary aspergillosis (IA) is a common infectious disease in patients with hematological diseases. The prevention, early detection, and establishment of treatment strategies for IA are important. The serum galactomannan antigen (GM) mycological test for IA diagnosis, included in the mycology criteria of the European Organization for Research and Treatment of Cancer-Invasive Fungal Infections Cooperative Group/National Institute of Allergy and Infectious Diseases Mycosis Study Group (EORTC/MSG), is widely used because of its high sensitivity and specificity. However, false-positive results are a concern. Methods: We retrospectively analyzed all GM tests performed at our department in the clinical practice setting between April 2003 and January 2012. Results: Of the 330 cases and 2155 samples analyzed, 540 (25%) were positive (≥0.5). Among the underlying diseases, positivity rates were the highest for multiple myeloma (MM), with 61.3%. By type, positivity rates for IgG, IgA, Bence-Jones protein, and IgD were 71.7%, 33.3%, 57.1%, and 34.6%, respectively. Seventeen out of eighteen cases that were GM-positive at MM diagnosis were false positives, according to the 2008 EORTC/MSG criteria. The IgG and GM values were not directly correlated. Of the seventeen false-positive cases identified, two developed IA during anti-myeloma treatments, and GM values did not become negative during the treatment in most cases. Conclusions: Although subclinical IA may be included in a higher GM index, the results may be prone to false positives; particularly in IgG-type MM, the results should thus be interpreted cautiously.

## Simple Summary

Invasive pulmonary aspergillosis (IA) is a common infectious disease in patients with hematological diseases. One of the mycological tests available for IA diagnosis is the serum galactomannan (GM) antigen test. However, GM tests sometimes yield false-positive results. We retrospectively reviewed 330 cases of hematological diseases and found that multiple myeloma, particularly the IgG type, was prone to false-positive GM test results. Although the higher GM test for myeloma may include subclinical IA, the results should be interpreted with caution.

## 1. Introduction

Invasive aspergillosis (IA) is a common infectious complication in patients with hematological diseases, such as acute leukemia or lymphoid malignancies. Prolonged neutropenia and the administration of high-dose corticosteroids represent classical risk factors for the development of IA. Moreover, the receipt of an allogeneic stem cell transplant; receipt of a solid organ transplant; treatment with other recognized T-cell immunosuppressants, such as calcineurin inhibitors, tumor necrosis factor-α blockers, lymphocyte-specific monoclonal antibodies, and immunosuppressive nucleoside analogs during the past 90 days; treatment with recognized B-cell immunosuppressants, such as Bruton’s tyrosine kinase inhibitors; severe combined immunodeficiency; and acute graft-versus-host disease were included as host factors in the 2008–2020 European Organization for Research and Treatment of Cancer-Invasive Fungal Infections Cooperative Group/National Institute of Allergy and Infectious Diseases Mycosis Study Group (EORTC/MSG) criteria [1,2].

IA remains a life-threatening complication of many treatments for hematological diseases. It tends to develop during induction therapies for leukemia or stem cell transplantation. In a previous study, IA had developed in 18 (1.5%) patients out of 1188 who received autologous stem cell transplantations [3]. The incidence of IA was reported as 42 (6.2%) in 669 patients with multiple myeloma (MM) in another study [4]. In a cohort of patients with hematological malignancies who developed IA, the mortality attributable to IA was high (9%), even though the patients were appropriately treated with voriconazole [5]. Therefore, early and accurate diagnosis remains critical for IA; however, this has not been clearly established.

The *Aspergillus* galactomannan (GM) antigen test represents one of the major mycological methods used for diagnosing IAs, and is included in mycological tests listed by the EORTC/MSG. The GM test has been reported to have excellent sensitivity and specificity. Its cutoff value in serum or plasma was determined as 0.5, according to the EORTC/MSG 2008 guidelines, and 1.0 for single-serum or plasma samples, according to the 2020 revision [2] (Table S1). The serum GM test is a simple laboratory test that is clinically useful, but several factors can influence its results [6,7,8,9,10]. Therefore, GM test results should be interpreted cautiously. Only a few large-scale epidemiological studies have assessed this controversy in MM among the hematological malignancies [11,12,13]. In addition, the clinical courses of false-positive GM tests remain poorly understood.

We conducted a large-scale retrospective study at our hospital to investigate the clinical utility and false-positive rates of GM tests for hematological diseases, including MM.

## 2. Materials and Methods

### 2.1. Study Design, Participants, and Ethics Statement

This retrospective study used information obtained from our hospital’s electronic medical records (Tokushima University Hospital, Tokushima, Japan). We included patients whose serum GM test results during routine clinical practice were obtained between April 2003 and January 2012. Clinical patient data (age, sex, underlying disease, hematological disease treatments, imaging data, and antifungal treatments) were retrospectively analyzed using electronic medical charts. The diagnostic criteria for IA were based on the 2008 or 2020 revisions of the EORTC/MSG criteria [1,2]. This study was conducted in accordance with the Declaration of Helsinki and approved by the institutional review board of the Tokushima University Ethics Committee (permission number: 2202-4).

### 2.2. Serum Galactomannan Test

Patient sera were collected during routine medical practices, and all GM tests were outsourced and performed at SRL Inc. (Tokyo, Japan).

### 2.3. Galactomannan Test in Paraproteins

We collected serum samples from patients with MM whose GM test results were false positive, according to the EORTC/MSG 2008 criteria. IgG was purified using a spin column-based antibody purification kit (Cosmo Bio Inc., Tokyo, Japan), according to the manufacturer’s instructions. GM tests were performed on the serum samples both before and after IgG purification.

### 2.4. Statistical Analysis

Continuous variables with normal distributions were expressed as means ± standard deviations, and those with non-normal distributions were expressed as medians (first quartile [Q1], third quartile [Q3]). Fisher’s exact and Student’s *t*-tests were performed using EZR software Ver.1.68 (Saitama Medical Center, Jichi Medical University, Saitama, Japan) [14].

## 3. Results

### 3.1. Patient Characteristics

A total of 330 eligible patients were included in this study, with 2155 samples analyzed. Of all the patients, 77 (23.3%) had MM and 74 (22.4%) had diffuse large B-cell lymphoma; the other diseases identified are shown in Table 1. Among the 2155 samples, the rates of positive (≥0.5 or ≥1.0) GM tests were 540 (25%) and 235 (10.9%), respectively. The highest positivity rate across various hematological diseases was observed for MM, which was ≥0.5 and ≥1.0 of 279 (61.3%) and 111 (24.3%) samples, respectively. Conversely, the lowest rate observed was for chronic myeloid leukemia, which had a ≥0.5 positivity for seven (4.8%) samples. Although the timing and/or treatment types were inconsistent among these samples, the positivity rate of GM in MM was extremely high.

### 3.2. Galactomannan Index in Hematological Diseases

**Figure 1:**
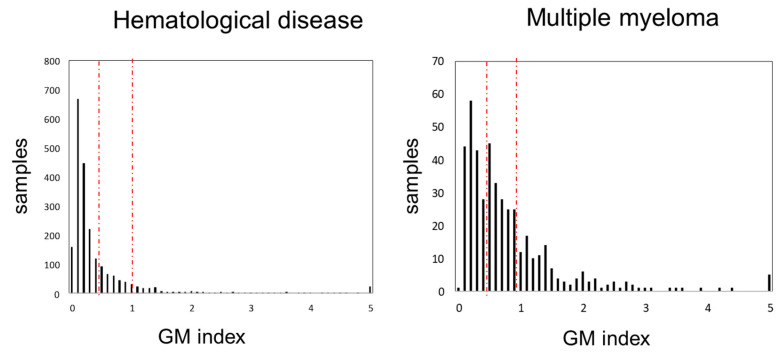
Distribution of GM index values. GM index distribution in all hematological diseases (**left**) and multiple myeloma (**right**). Left and right red dash line indicate over 0.5, 1.0, respectively. Abbreviations: GM, *Aspergillus* galactomannan.

We compared the frequency distribution of GM antigen test values among all hematological diseases and MM (Figure 1). The peak values were 0.1 for all hematological diseases and 0.2 for MM. The second peak in MM was observed at 0.5. As the GM index value increased, the frequency tended to decrease; however, some cases with values of 5.0 were observed. The median GM index values for all hematological diseases and MM were 0.2 (0.1–0.5) and 0.6 (0.3–1.0), respectively (*p* = 1.31 × 10^−17^).

### 3.3. Galactomannan Index in Multiple Myeloma

We then investigated the GM index values in 77 patients with MM (453 samples). When these were classified by M protein type (the distribution is shown in Figure S1), of the 283 samples from patients with IgG-type MM, 202 (69.5%) had positivity rates ≥0.5 and 91 (20.0%) had positivity rates >1.0. For IgA-type MM (36 samples), twelve (33.3%) had positivity rates ≥0.5 and eight (22.2%) had positivity rates >1.0. Of the 79 samples obtained from patients with Bence-Jones protein (BJP)-type MM, 46 (58.2%) and 19 (24.0%) had positivity rates ≥0.5 and >1.0, respectively. In IgD-type MM (53 samples), nineteen samples (35.8%) had positivity rates ≥0.5 and five (9.4%) had positivity rates ≥1.0 (Figure S1). More GM-positive samples in the IgG type were observed than in the IgA, BJP, and IgD types; however, these GM test results included measurements taken during the administration of systemic chemotherapy in the majority of patients, as well as during episodes of clinical signs, such as fever. Therefore, we investigated the limitations of GM testing in newly diagnosed patients with MM prior to systemic chemotherapy to minimize confounding factors. GM indexes were measured prior to the first chemotherapy in about two-thirds of patients with newly diagnosed MM and in 28 of 44 patients with IgG-type newly diagnosed MM. The distribution by subgroup is shown in Figure S2. The patients were not administered antibiotics, enteral nutrition, or intravenous immunoglobulin. Among the 28 patients with IgG-type MM, 18 were GM-positive and 10 GM-negative. This IgG type had the highest positivity rate (Figure S2). Finally, 23 patients with IgG, IgA, BJP, and IgD types were analyzed using the GM test and determined to be positive (≥0.5) at the diagnosis of MM and before systemic chemotherapy. GM tests were performed for all 182 samples of these 23 patients (Table 2). For these 23 patients, the median GM index was higher than that of other GM tests performed during MM treatment (1.11 [0.6–1.4] vs. 0.61 [0.2–0.7]). The number of samples with a GM index ≥ 0.5 (≥0.5 or ≥1.0) at diagnosis of the MM group was greater than that when the GM index was measured during the treatment of the MM group: 155 (85.2%) vs. 124 (45.8%) and 76 (41.8%) vs. 47 (17.3%), respectively (Table 2).

### 3.4. Clinical Course of the Galactomannan Index for Positive IgG-Type Multiple Myeloma

**Figure 2:**
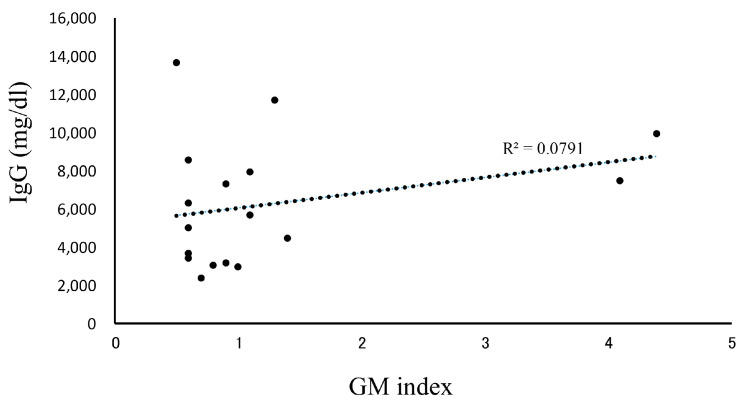
Correlation between IgG and the GM index. Patients with IgG-type multiple myeloma whose GM index was positive (≥0.5) and who were diagnosed as pseudo-positive. A total of 17 patients were analyzed for the correlation between IgG and the GM index. Abbreviations: GM, *Aspergillus* galactomannan.

**Figure 3:**
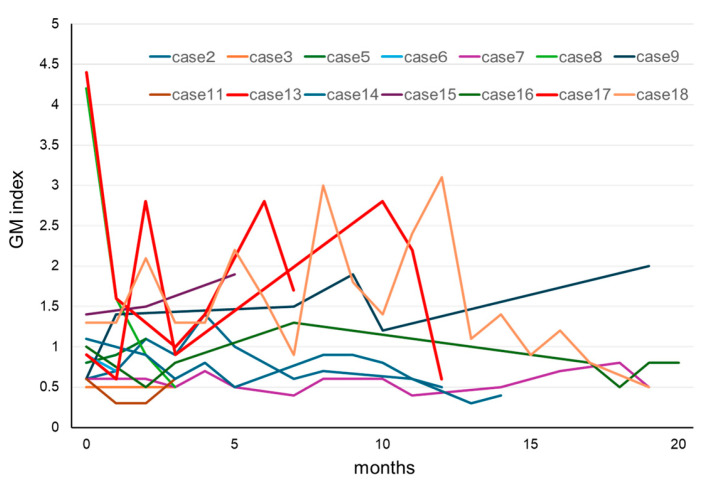
Clinical course of GM in patients with MM for whom the GM test results were positive at initial diagnosis of MM. In most cases, the GM index did not return to normal values during chemotherapy for MM. Cases 13 and 17 developed invasive aspergillosis during the clinical course. Abbreviations: GM, *Aspergillus* galactomannan and MM, multiple myeloma.

In our patients who tested positive for GM at initial diagnosis of MM, the effects of treatment were excluded, and the influence of confounding factors was assumed to be negligible. Therefore, we decided to further examine our 18 cases of IgG-type MM who had positive GM test results at their initial examinations (Table 3). Their ages were in the range of 50–79 years, and no statistically significant differences were observed in terms of sex. The GM index varied between a relatively low value of 0.6 and a high one of 4.1. The clinical factors diagnosed via imaging were positive in one patient who was diagnosed with probable IA, according to the 2008 EORTC/MSG criteria. A correlation between IgG and the GM index was not observed in 17 false-positive cases, other than in one probable case (Figure 2). We retrospectively assessed the subsequent GM indexes in these cases. When we analyzed the 14 patients that were followed up for >3 months, we found that most of their GM indexes remained in the positive range of ≥0.5 (Figure 3). Preventive oral medications with anti-*Aspergillus* activities, as well as itraconazole, were administered in nine of the cases, excluding cases 5, 6, 8, 14, and 15; however, their GM index scores did not differ substantially. Patients 13 and 17 developed IA while receiving vincristine, adriamycin, and high-dose dexamethasone therapy.

### 3.5. Galactomannan Index in Paraprotein

We collected four serum samples from patients in the group with IgG-type MM, with GM indexes ≥0.5 that were considered false positives, and measured the GM indexes of the samples before and after IgG purification. Although it was not possible to purify all paraproteins, due to the large amount of IgG present, the GM index was found to correlate with IgG both in the raw samples and after purification. The M protein was identified as the main component, and the GM/IgG ratio was approximately the same as that of the samples measured before paraprotein purification (Figure S3).

## 4. Discussion

This study retrospectively examined the clinical significance of the *Aspergillus* GM test used in our department, with a focus on hematological diseases. MMs, particularly of the IgG type, tend to have relatively high serum GM index values.

### 4.1. False-Positive or Negative Galactomannan Test Results

The GM test is generally performed using the Platelia *Aspergillus* enzyme-linked immunosorbent assay (ELISA) with the anti-GM monoclonal antibody, EB-A2, which binds to an epitope composed of four β(1–5)-linked galactofuranose residues [15]. Its clinical utility was reviewed in this study, and its overall sensitivity and specificity values were found to be 78% (61–89%) and 81% (72–88%), respectively, when an optical density index (ODI) of 0.5 was used as the cutoff value [16]. However, many factors can contribute to false-positive GM test results, including drugs (e.g., antimicrobials, such as intravenous tazobactam or piperacillin [17], amoxicillin [18], and inhaled colistin [10]), infectious diseases (e.g., histoplasmosis [19], nocardiosis [20], fusariosis [7], and cryptococcosis [21]), and fungal invasion through the intestinal mucosa (such as during graft-versus-host disease [22], enteral feeding [23], aspiration pneumonia [24], and the administration of intravenous immunoglobulin [9]). The mechanisms by which these factors increase the GM index likely involve cross-reactions with IgG antibodies specific to EB-A2.

Conversely, prophylactic antifungal medications have been reported to decrease the GM index and cause more false-negative results [25]. In one report, a total of 272 serum samples were obtained from 46 patients with IA, and 3005 serum samples were obtained from 269 control patients. The sensitivity of the GM test to proven or probable IA was reduced from 89% (65–97%) to 52% (32–71%) when a cutoff value of 0.5 was used within one week of IA development [25]. Therefore, caution should be exercised when interpreting the GM test results.

### 4.2. Positivity of the Galactomannan Test in Multiple Myeloma

Higher GM test positivity rates have been reported in patients with MM among those with hematological diseases [11,12,13]. Mori et al. reported false-positive GM test results in 11 patients with MM [11]. Ko et al. investigated 30 false-positive and 316 negative cases in patients with cancer. Among all the related factors, they found that MM was the only significant one (odds ratio, 3.59; 95% confidence interval, 1.28–10.04). Among their cohort of 145 patients with MM, 25.5% showed false-positive results, which was three times higher than when the GM test cutoff value was set to 0.5 [12]. Abe et al. reported an association between false-positive serum GM test results and age among 1071 healthy adult subjects. The GM test results correlated with serum IgG levels in 700 patients with newly diagnosed hematological diseases, including patients with MM and without probable or proven IA. Age- and/or disease-related elevations in IgG levels may, therefore, cause false-positive GM test results [13]. Our study showed higher GM test positivity among patients with MM, even for those with newly diagnosed MM who were not administered steroids or other chemotherapeutic drugs. These data are consistent with those of a previous study [11,13]. An association between the IgG levels and the GM index was not observed in our study (Figure 2). However, the higher positivity of the GM test results in patients with MM observed in this study may be partially attributed to the higher testing rate of GM in our clinical setting compared with that in other hospitals.

### 4.3. Transition of the Galactomannan Test Cutoff Value

The cutoff value of the GM index was previously set to 1.5. However, its sensitivity was low in patients with neutropenia; therefore, this cutoff value was lowered to 0.5 based on the results of several studies [26,27]. One meta-analysis [28] analyzed GM results in several studies that used different ODI cutoff values. In seven studies (901 total patients) that used an ODI of 0.5 as the cutoff value, the overall sensitivity was 78% (61–89%) and the specificity was 81% (72–88%) to diagnose proven or probable IA. When 12 studies (1744 total patients) that used a cutoff ODI value of 1.0 were analyzed, the overall sensitivity of the GM test was reported to be 75% (59–86%), and its mean specificity 91% (84–95%). In 17 studies (2600 total patients) that used a 1.5 ODI cutoff value, the sensitivity was 64% (50–77%) and the mean specificity 95% (91–97%) [28]. An updated meta-analysis showed that the sensitivity of the test was 0.78 (0.70–0.85) and its specificity 0.85 (0.78–0.91), including in studies with 394 patients with proven or probable IA and 3549 with possible or no IA at a cutoff value of 0.5. When using a cutoff value of 1.0, the sensitivity was 0.71 (0.63–0.78) and the specificity was 0.90 (0.86–0.93) in a study of 145 patients with proven or probable IA and 1246 with possible or no IA. In a study of 209 patients with proven or probable IA and 2412 with possible or no IA, the sensitivity was 0.63 (0.49–0.77) and the specificity was 0.93 (0.89–0.97) when the cutoff value was 1.5 [16]. Given these data, the cutoff value of the GM test in the 2020 EORTC/MSG criteria was set to 1.0 [2]. In our cohort, the seventeen patients with newly diagnosed MM who had false-positive GM test results (cutoff ≥0.5, according to the 2008 EORTC/MSG 2008 criteria) would have been reduced to seven if we had applied the 2020 EORTC/MSG criteria (cutoff ≥ 1.0).

### 4.4. Mechanisms of False-Positive Galactomannan Test Results in Multiple Myeloma

The mechanisms underlying the false elevation of the GM index in patients with IgG-type MM have not yet been elucidated. The GM index was measured using a sandwich ELISA with the EB-A2 IgG-type monoclonal antibody [15]. This showed that high serum IgG levels may possibility cross-react with GM-specific IgG antibodies. Additionally, we purified the M protein from the serum samples of patients and subsequently measured the GM index. These results showed a correlation between the IgG levels and GM indexes both before and after M protein purification. Therefore, part of the GM may bind to IgG. In Figure 2, the direct association in vivo between the GM test results and IgG levels was poor. This is likely because the amount of M protein and severity of MM vary from person to person. One study measured the association between non-paraprotein IgG levels and the GM index in healthy volunteers [13]. Therefore, some normal IgGs may bind to the GM antigen; however, IgG-type paraproteins may bind more. Paraproteins have been reported to bind to a range of proteins. IgA-type paraproteins have been reported to bind to protein A [28] and protein L, a bacterial protein, binding to the light chain [29]. Other proteins have been shown to bind to the heavy chain [30]. Although clarifying whether GM binds to the M protein is difficult, this represents one of the factors that affects the results of various tests.

One of the patients in our study with IgG-type newly diagnosed MM who had a positive (≥0.5) GM test result had probable IA. Two of our sixteen patients with IgG-type MM developed IA during follow-up. These data suggest that a higher GM index may detect not only cross-reactions in assays, but also subclinical IA. In cases of proven or probable IA, a decrease in the GM index is a useful indicator of an early response and positive outcome [31,32]. However, in most of our patients with MM, the GM index did not decrease to within the normal range (<0.4), regardless of treatment for MM. Mori et al. reported persistent false-positive GM tests in eleven patients with MM, of whom eight remained positive (≥0.5) until the end of the study [11]. In cases of false-positive GM test results in patients with MM, the GM test may be unreliable; therefore, careful clinical judgment and/or the use of other tests, such as PCR, may be warranted in clinical practice. The sensitivity and specificity values of PCR tests for diagnosing IA vary (58–80.5% and 78.5–95.2%, respectively) depending on the criteria used to define a positive test [33]. Another study demonstrated a unique approach to the early identification of IA by combining the weekly GM test and chest CT results for early IA detection, even in patients who were administered prophylactic antifungal medications or had false-positive GM test results [34].

### 4.5. Limitations

This study was subject to several key limitations. This was a retrospective study wherein we collected and summarized GM test results acquired during clinical practice. Therefore, serum collection for the GM test was not performed in a uniform manner. Patients with higher GM test scores tended to have follow-up GM tests, which raises some concerns regarding the frequency of positive GM test results. Most of the patients with MM assessed in our study were of the IgG type, and analyses of IgA, IgD, and BJP types were insufficient. In addition, we have not prospectively validated our observations in an independent cohort of MM. As bortezomib was approved for newly diagnosed MM in 2011 in Japan, most of the patients in our cohort with newly diagnosed MM were treated with high-dose dexamethasone-based therapy with or without cytotoxic chemotherapeutic drugs, rather than with novel drugs, such as bortezomib. Therefore, the standard treatment regimen used during our study, including the corticosteroid dose, was completely different from the current standard treatment regimen. To date, many anti-filamentous fungal drugs have been approved, and preventive medication techniques and treatment environments in hospitals have progressed considerably in developed countries. Therefore, the incidence of IA in our cohort likely differs from that in current patients with MM.

## 5. Conclusions

The GM index tends to be high in patients with MM who have undergone mycological testing, particularly for the IgG type, which is prone to persistent false-positive results. Therefore, GM test results should be interpreted with caution. However, some patients may develop IA even if their GM test results are judged as false positives. Therefore, subclinical IA may be included in a higher GM index. Distinguishing between subclinical IA and a false positive based on GM test results is currently impossible. In IgG-type MM, other tests or combinations, such as PCR tests and/or chest CT, may be useful for accurate diagnosis and monitoring. The development of more specific tests for IA is warranted.

## Figures and Tables

**Table 1:** Patients analyzed for *Aspergillus* galactomannan antigen.

	Patients	Samples	Positive (≥0.5) Samples	Positive (≥0.5) Rate	Positive (≥1.0) Samples	Positive (≥1.0) Rate
Total	330	2155	540	25%	235	10.9%
MM	77	453	279	61.3%	111	24.5%
DLBCL	74	313	63	20%	29	9.3%
AML	52	533	54	10.1%	23	4.3%
T-cell lymphoma	16	73	5	6.8%	3	4.1%
FL	12	51	15	29%	4	7.8%
ALL	12	91	6	6.6%	0	0
AA	12	75	5	6.7%	4	5.3%
CML	10	145	7	4.8%	2	1.4%
MDS	10	70	14	20%	6	8.6%
ATL	9	56	5	8.9%	0	0
MCL	6	61	23	37.7%	12	19.7%
Others	40	234	64	27.6%	61	26.1%

Abbreviations: MM, multiple myeloma; DLBCL, diffuse large B-cell lymphoma; AML, acute myeloid leukemia; FL, follicular lymphoma; ALL, acute lymphoblastic leukemia; AA, aplastic anemia; CML, chronic myeloid leukemia; MDS, myelodysplastic neoplasm; ATL, adult T-cell leukemia; and MCL, mantle cell lymphoma.

**Table 2:** Galactomannan index in patients with multiple myeloma.

	Patients with MM (*N* = 77)	Positive GM Index (≥0.5) at Diagnosis of MM (*N* = 23)	GM Index Measured During MM Treatment (*N* = 54)
Samples	453	182	271
Age—years, median (IQR)	64 (58.0–71.0)	65 (61.5–69.5)	64 (54.25–71)
Female sex, -*n* (%)	35 (45.5%)	9 (39.1%)	26 (48.1%)
Type			
IgG, -*n* (%)	44 (57.1%)	18 (78.3%)	26 (48.1%)
IgA, -*n* (%)	12 (15.6%)	1 (4.3%)	11 (20.4%)
BJP, -*n* (%)	13 (16.9%)	2 (8.7%)	11 (20.4%)
IgD, -*n* (%)	7 (9.1%)	2 (8.7%)	6 (11.1%)
Nonsecretory, -*n* (%)	1 (1.3%)	0 (0.0%)	1 (1.9%)
GM index median (IQR)	0.82 (0.3–1.0)	1.11 (0.6–1.4)	0.61 (0.2–0.7)
GM index ≥ 0.5 samples, (%)	279 (61.6%)	155 (85.2%)	124 (45.8%)
GM index ≥ 1.0 samples, (%)	111 (24.5%)	76 (41.8%)	47 (17.3%)

Abbreviations: GM, *Aspergillus* galactomannan; MM, multiple myeloma; and IQR, interquartile range.

**Table 3:** Patients in whom the galactomannan test was positive (≥0.5) at the initial diagnosis of multiple myeloma.

Case	Age·Sex	GM Index	IgG	Host Factor	Clinical Factor	Diagnosis (EORTC/MSG 2008)	Diagnosis (EORTC/MSG 2020)	Initial Treatment
1	63F	0.6	4988	Hematologic malignancy (multiple myeloma)	−	False-positive	No IFD	VAD
2	65M	0.6	3429		−	False-positive	No IFD	HDD
3	61M	0.5	13629		−	False-positive	No IFD	HDD
4	64F	1.1	5661		−	False-positive	False-positive	HDD
5	72M	0.8	3042		−	False-positive	No IFD	BD
6	77M	0.9	7286		−	False-positive	No IFD	MP
7	72M	0.6	6308		−	False-positive	No IFD	MP
8	64F	4.1	7451		−	False-positive	False-positive	BD
9	57M	0.6	8565		−	False-positive	No IFD	VAD
10	73F	0.8	4292		+ ^※^	Probable	No IFD	VAD
11	63M	0.6	3655		−	False-positive	No IFD	BD
12	64F	0.7	2379		−	False-positive	No IFD	HDD
13	61F	4.4	9928		−	False-positive	False-positive	HDD
14	70M	1.1	7923		−	False-positive	False-positive	MP
15	69M	1.4	4441		−	False-positive	False-positive	MP
16	51M	1.0	2939		−	False-positive	False-positive	VAD
17	70F	0.9	3167		−	False-positive	No IFD	VAD
18	66F	1.3	11674		−	False-positive	False-positive	VAD

^※^ Wedge-shaped and segmental or lobar consolidation via computed tomography. Abbreviations: GM, *Aspergillus* galactomannan; M, male; F, female; IFD, invasive fungal disease; EORTC/MSG, European Organization for Research and Treatment of Cancer Invasive Fungal Infections Cooperative Group/National Institute of Allergy and Infectious Diseases Mycosis Study Group; VAD, vincristine, adriamycin, dexamethasone; HDD, high-dose dexamethasone; BD, bortezomib, dexamethasone; and MP, melphalan, prednisolone.

## Data Availability

The data analyzed in this study will be made available upon reasonable request by the corresponding author.

## Supplementary Materials

The following supporting information can be downloaded at: https://www.mdpi.com/article/10.3390/diseases13040118/s1. Figure S1. Number of positive (≥0.5) and negative (<0.4) samples in MM by type; Figure S2. Number of positive samples measured for the GM index in MM by type at initial diagnosis and before chemotherapy against MM; Figure S3. Four serum samples of IgG-type MM obtained from patients with GM indexes ≥ 0.5 that were considered false positives, and the GM indexes of the samples measured before and after IgG purification. IgG was purified using a spin column-based antibody purification kit (Cosmo Bio Inc., Tokyo, Japan), according to the manufacturer’s instructions; Table S1. Mycological evidence of aspergillosis with an indirect test using EORTC/MSG criteria.

## References

[1] De Pauw B., Walsh T.J., Donnelly J.P., Stevens D.A., Edwards J.E., Calandra T., Pappas P.G., Maertens J., Lortholary O., Kauffman C.A. (2008). Revised definitions of invasive fungal disease from the European Organization for Research and Treatment of Cancer/Invasive Fungal Infections Cooperative Group and the National Institute of Allergy and Infectious Diseases Mycoses Study Group (EORTC/MSG) Consensus Group. Clin. Infect. Dis..

[2] Donnelly J.P., Chen S.C., Kauffman C.A., Steinbach W.J., Baddley J.W., Verweij P.E., Clancy C.J., Wingard J.R., Lockhart S.R., Groll A.H. (2020). Revision and Update of the Consensus Definitions of Invasive Fungal Disease From the European Organization for Research and Treatment of Cancer and the Mycoses Study Group Education and Research Consortium. Clin. Infect. Dis..

[3] Jantunen E., Salonen J., Juvonen E., Koivunen E., Siitonen T., Lehtinen T., Kuittinen O., Leppä S., Anttila V.J., Itälä M. (2004). Invasive fungal infections in autologous stem cell transplant recipients: A nation-wide study of 1188 transplanted patients. Eur. J. Haematol..

[4] Fuchs E., Lavi N., Carasso Y., Oren I., Hardak E. (2021). Invasive Pulmonary Aspergillosis in Multiple Myeloma patients: A sizeable diagnosis in the era of novel anti-myeloma therapies. Mycoses.

[5] Hachem R., Batista M.V., Kanj S.S., El Zein S., Haddad S., Jiang Y., Mori N., Vanderson R., Chaftari A.M., Raad I. (2019). International Multicenter Experience in the Treatment Outcome of Invasive Aspergillosis in Immunocompromised Cancer Patients. Mediterr. J. Hematol. Infect. Dis..

[6] Mikulska M., Furfaro E., Del Bono V., Raiola A.M., Ratto S., Bacigalupo A., Viscoli C. (2012). Piperacillin/tazobactam (Tazocin) seems to be no longer responsible for false-positive results of the galactomannan assay. J. Antimicrob. Chemother..

[7] Tortorano A.M., Esposto M.C., Prigitano A., Grancini A., Ossi C., Cavanna C., Cascio G.L. (2012). Cross-reactivity of Fusarium spp. in the Aspergillus Galactomannan enzyme-linked immunosorbent assay. J. Clin. Microbiol..

[8] Ng T.Y., Kang M.L., Tan B.H., Ngan C.C. (2014). Case report: Enteral nutritional supplement as a likely cause of false-positive galactomannan testing. Med. Mycol. Case Rep..

[9] Liu W.D., Lin S.W., Shih M.C., Su C.L., Wang Y.W., Lin S.C., Lee Y.F., Huang H.H., Chou W.C., Wu U.I. (2020). False-positive Aspergillus galactomannan immunoassays associated with intravenous human immunoglobulin administration. Clin. Microbiol. Infect..

[10] Hung Y.H., Lai H.H., Lin H.C., Sun K.S., Chen C.Y. (2021). Investigating Factors of False-Positive Results of Aspergillus Galactomannan Assay: A Case-Control Study in Intensive Care Units. Front. Pharmacol..

[11] Mori Y., Nagasaki Y., Kamezaki K., Takenaka K., Iwasaki H., Harada N., Miyamoto T., Abe Y., Shimono N., Akashi K. (2010). High incidence of false-positive Aspergillus galactomannan test in multiple myeloma. Am. J. Hematol..

[12] Ko J.H., Peck K.R., Lee J.Y., Cho S.Y., Ha Y.E., Kang C.I., Chung D.R., Kim K., Kang E.S., Song J.H. (2016). Multiple myeloma as a major cause of false-positive galactomannan tests in adult patients with cancer. J. Infect..

[13] Abe Y., Narita K., Kobayashi H., Kitadate A., Takeuchi M., Matsue K. (2019). Higher frequency of false-positive serum galactomannan tests among older subjects and the association with elevated serum immunoglobulin G levels. Mycoses.

[14] Kanda Y. (2013). Investigation of the freely available easy-to-use software ‘EZR’ for medical statistics. Bone Marrow Transplant..

[15] Stynen D., Sarfati J., Goris A., Prevost M.C., Lesourd M., Kamphuis H., Darras V., Latge J.P. (1992). Rat monoclonal antibodies against Aspergillus galactomannan. Infect. Immun..

[16] Leeflang M.M., Debets-Ossenkopp Y.J., Wang J., Visser C.E., Scholten R.J., Hooft L., Bijlmer H.A., Reitsma J.B., Zhang M., Bossuyt P.M. (2015). Galactomannan detection for invasive aspergillosis in immunocompromised patients. Cochrane Database Syst. Rev..

[17] Viscoli C., Machetti M., Cappellano P., Bucci B., Bruzzi P., Van Lint M.T., Bacigalupo A. (2004). False-positive galactomannan platelia Aspergillus test results for patients receiving piperacillin-tazobactam. Clin. Infect. Dis..

[18] Mattei D., Rapezzi D., Mordini N., Cuda F., Lo Nigro C., Musso M., Arnelli A., Cagnassi S., Gallamini A. (2004). False-positive Aspergillus galactomannan enzyme-linked immunosorbent assay results in vivo during amoxicillin-clavulanic acid treatment. J. Clin. Microbiol..

[19] Wheat L.J., Hackett E., Durkin M., Connolly P., Petraitiene R., Walsh T.J., Knox K., Hage C. (2007). Histoplasmosis-associated cross-reactivity in the BioRad Platelia Aspergillus enzyme immunoassay. Clin. Vaccine Immunol..

[20] Haran A., Temper V., Assous M., Bergel M., Chahanian N., Elinav H., Korem M. (2021). False-positive galactomannan antigen testing in pulmonary nocardiosis. Med. Mycol..

[21] Dalle F., Charles P.E., Blanc K., Caillot D., Chavanet P., Dromer F., Bonnin A. (2005). Cryptococcus neoformans Galactoxylomannan contains an epitope(s) that is cross-reactive with Aspergillus Galactomannan. J. Clin. Microbiol..

[22] Murashige N., Kami M., Kishi Y., Fujisaki G., Tanosaki R. (2005). False-positive results of Aspergillus enzyme-linked immunosorbent assays for a patient with gastrointestinal graft-versus-host disease taking a nutrient containing soybean protein. Clin. Infect. Dis..

[23] Girmenia C., Santilli S., Ballaro D., Del Giudice I., Armiento D., Mauro F.R. (2011). Enteral nutrition may cause false-positive results of Aspergillus galactomannan assay in absence of gastrointestinal diseases. Mycoses.

[24] Tomita Y., Sugimoto M., Kawano O., Kohrogi H. (2009). High incidence of false-positive Aspergillus galactomannan test results in patients with aspiration pneumonia. J. Am. Geriatr. Soc..

[25] Marr K.A., Laverdiere M., Gugel A., Leisenring W. (2005). Antifungal therapy decreases sensitivity of the Aspergillus galactomannan enzyme immunoassay. Clin. Infect. Dis..

[26] Maertens J., Verhaegen J., Lagrou K., Van Eldere J., Boogaerts M. (2001). Screening for circulating galactomannan as a noninvasive diagnostic tool for invasive aspergillosis in prolonged neutropenic patients and stem cell transplantation recipients: A prospective validation. Blood.

[27] Maertens J.A., Klont R., Masson C., Theunissen K., Meersseman W., Lagrou K., Heinen C., Crépin B., Van Eldere J., Tabouret M. (2007). Optimization of the cutoff value for the Aspergillus double-sandwich enzyme immunoassay. Clin. Infect. Dis..

[28] Biewenga J., Daus F., Modderman M.L., Bruin G.M. (1982). Binding of human IgA myeloma proteins to protein A. Evidence for different binding mechanisms. Immunol. Commun..

[29] Akerström B., Björck L. (1989). Protein L: An immunoglobulin light chain-binding bacterial protein. Characterization of binding and physicochemical properties. J. Biol. Chem..

[30] Haas I.G., Wabl M. (1983). Immunoglobulin heavy chain binding protein. Nature.

[31] Woods G., Miceli M.H., Grazziutti M.L., Zhao W., Barlogie B., Anaissie E. (2007). Serum Aspergillus galactomannan antigen values strongly correlate with outcome of invasive aspergillosis: A study of 56 patients with hematologic cancer. Cancer.

[32] Nouér S.A., Nucci M., Kumar N.S., Grazziutti M., Barlogie B., Anaissie E. (2011). Earlier response assessment in invasive aspergillosis based on the kinetics of serum Aspergillus galactomannan: Proposal for a new definition. Clin. Infect. Dis..

[33] Cruciani M., Mengoli C., Loeffler J., Donnelly P., Barnes R., Jones B.L., Klingspor L., Morton O., Maertens J. (2015). Polymerase chain reaction blood tests for the diagnosis of invasive aspergillosis in immunocompromised people. Cochrane Database Syst. Rev..

[34] Wang X., Guo G., Cai R., He P., Zhang M. (2019). Utility of serum galactomannan antigen testing combined with chest computed tomography for early diagnosis of invasive pulmonary aspergillosis in patients with hematological malignancies with febrile neutropenia after antifungal drug treatment. J. Int. Med. Res..

