# Supplementary material for: False-Positive Galactomannan Test Results in Multiple Myeloma
Source: Diseases. 2025 Apr 17;13(4):118. doi: 10.3390/diseases13040118 (PMC12026058; doi:10.3390/diseases13040118)
Supplement: Supplementary file 1 [file diseases-13-00118-s001.zip › diseases-3547645-supplementary/myeloma aspergillus supplefigure 20250101.pptx]

## Slide 1
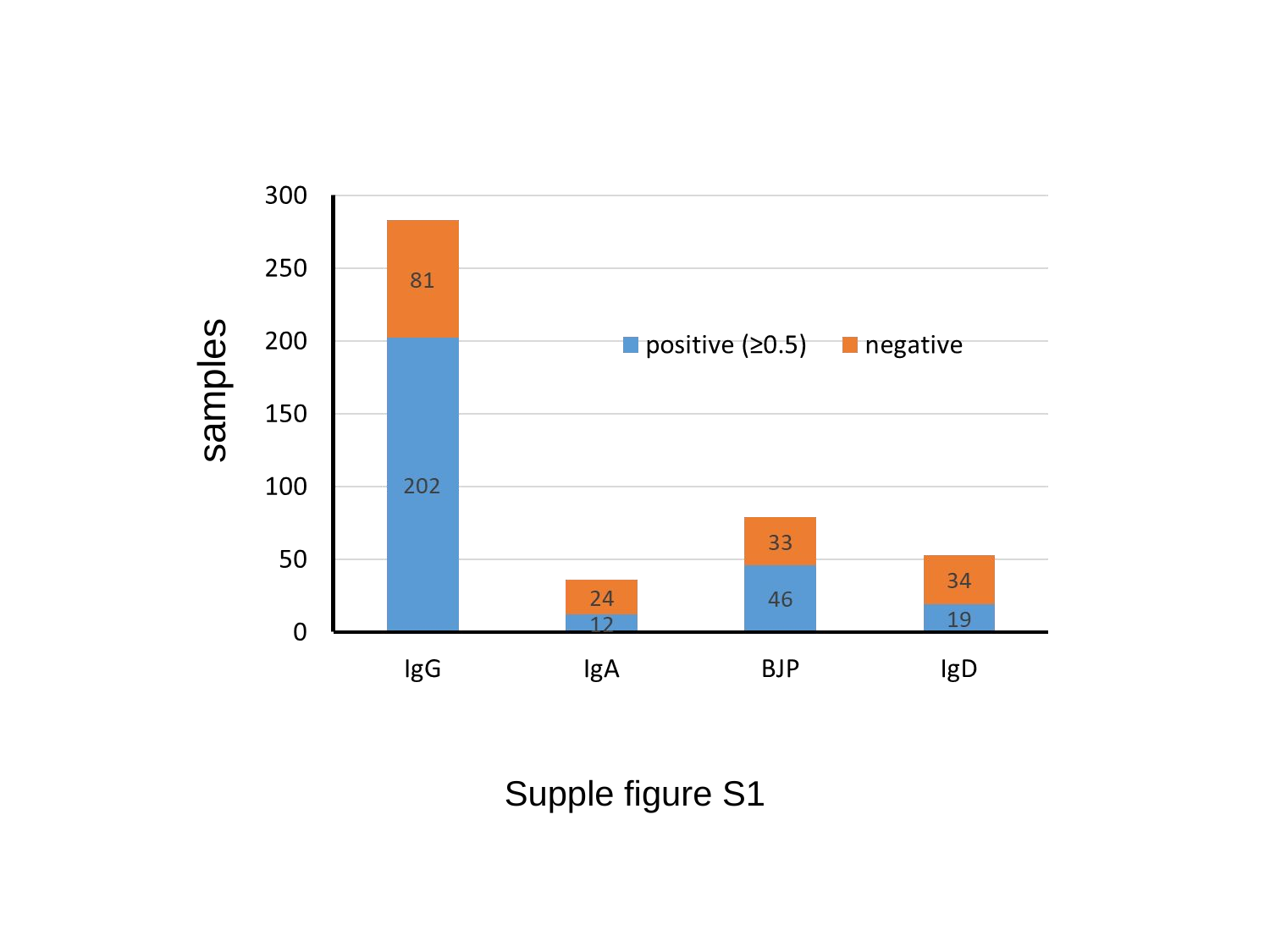

samples
# Supple figure S1

## Slide 2
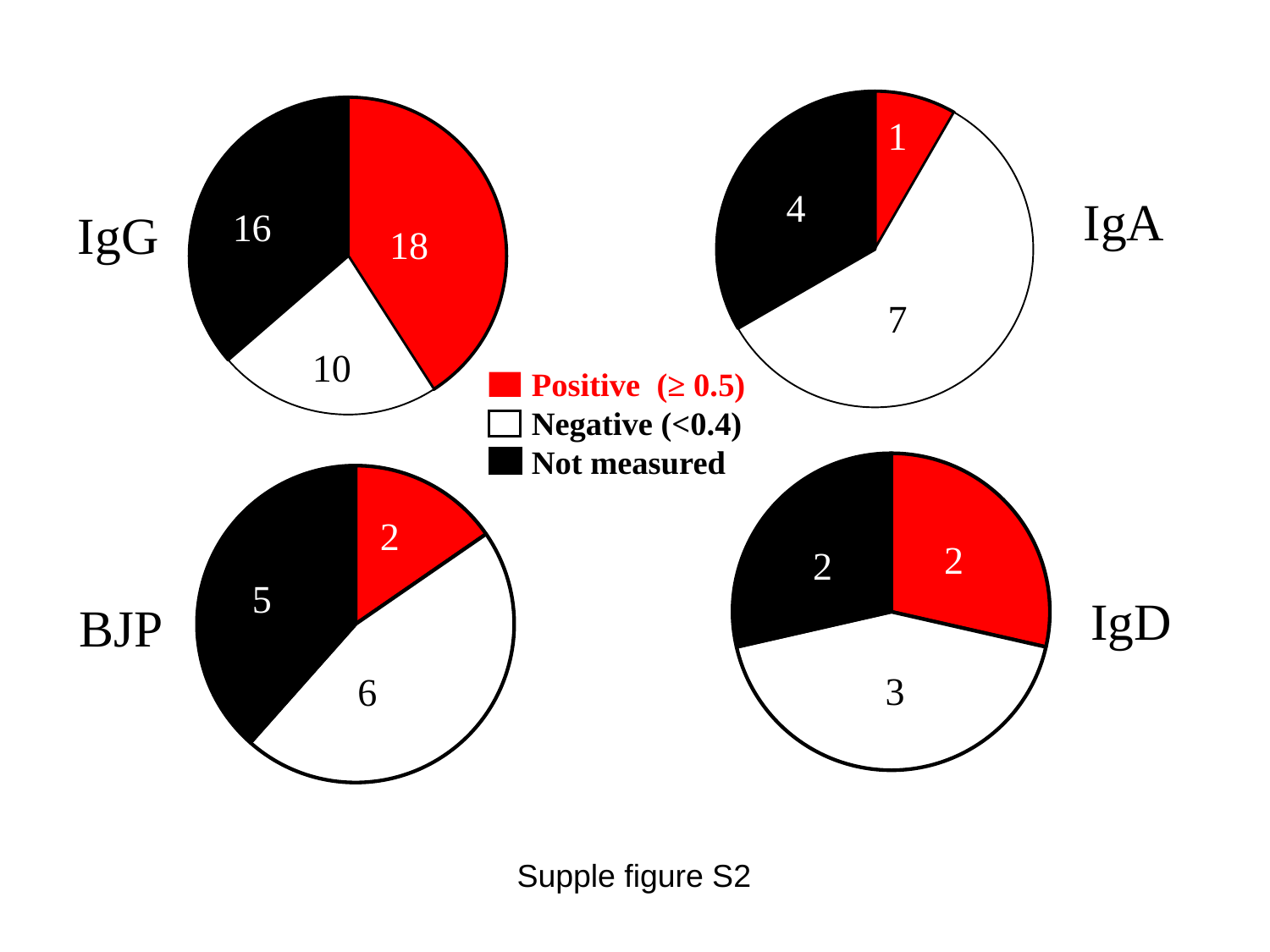

1
4
IgA
IgG
16
18
7
10
Positive (≥ 0.5)
Negative (<0.4)
Not measured
2
2
2
5
IgD
BJP
3
6
# Supple figure S2

## Slide 3
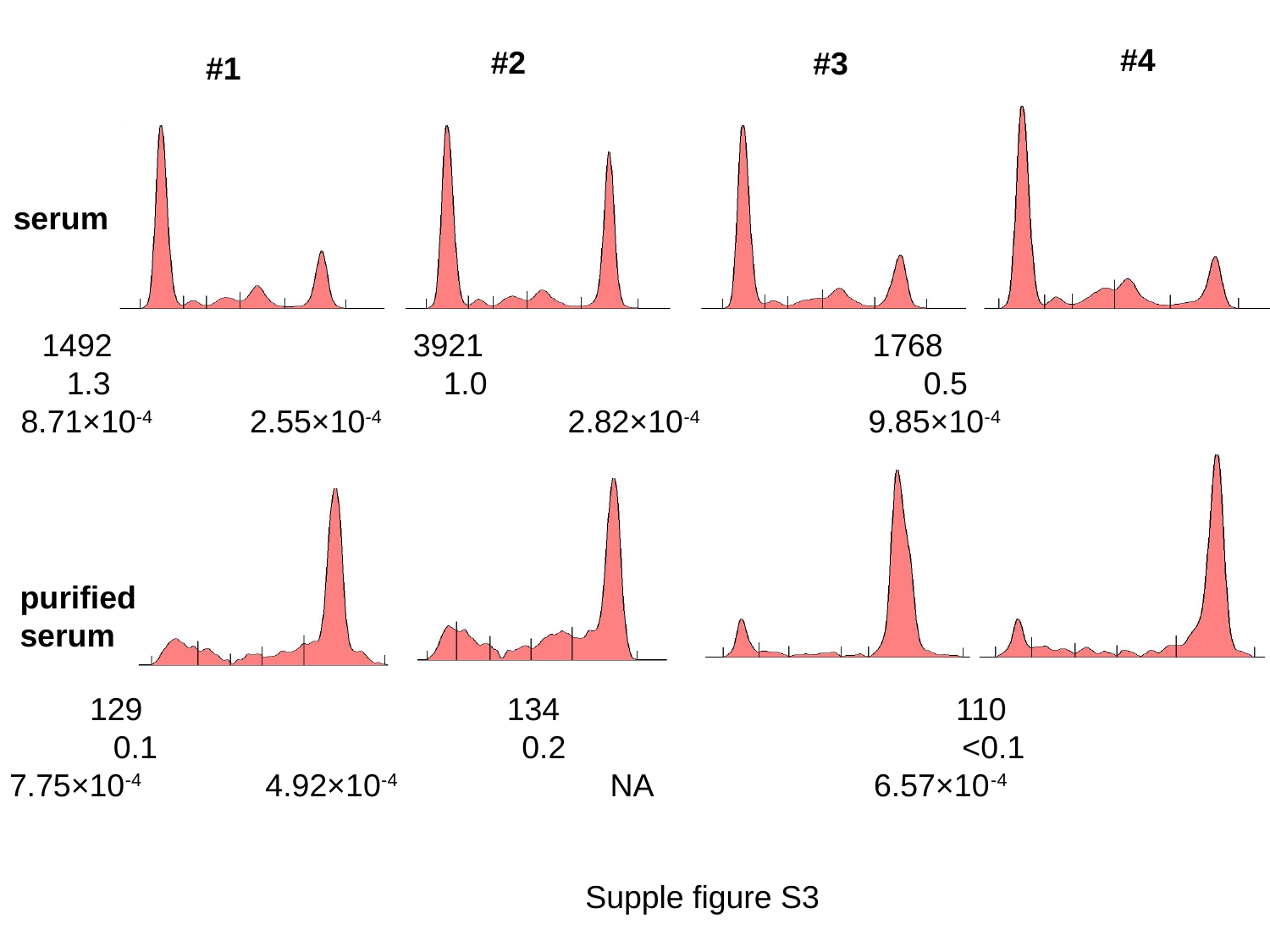

#4
#2
#3
#1
serum
IgG　　 1492　　　　　　　　　3921　　　　　　　　　　　 1768　　　　　　　　　　　　1218
GM　　 1.3　　　　　　　　　　1.0　　　　　　　　　　　　 　0.5　　　　　　　　 　　 　　1.2
GM/IgG　 8.71×10^-4^ 2.55×10^-4^ 2.82×10^-4^ 9.85×10^-4^
purified
serum
IgG　　　 129　　　　　　　　　　　134　　　　　　　　　　　　110　　　　　　　　　　　　152
GM　　　 　 0.1　　　　　　　　　　　0.2　　　　　　　　　　　　<0.1　　　　　　　　　　　　0.1
GM/IgG 7.75×10^-4^ 4.92×10^-4^ NA 6.57×10^-4^
Supple figure S3

## Slide 4
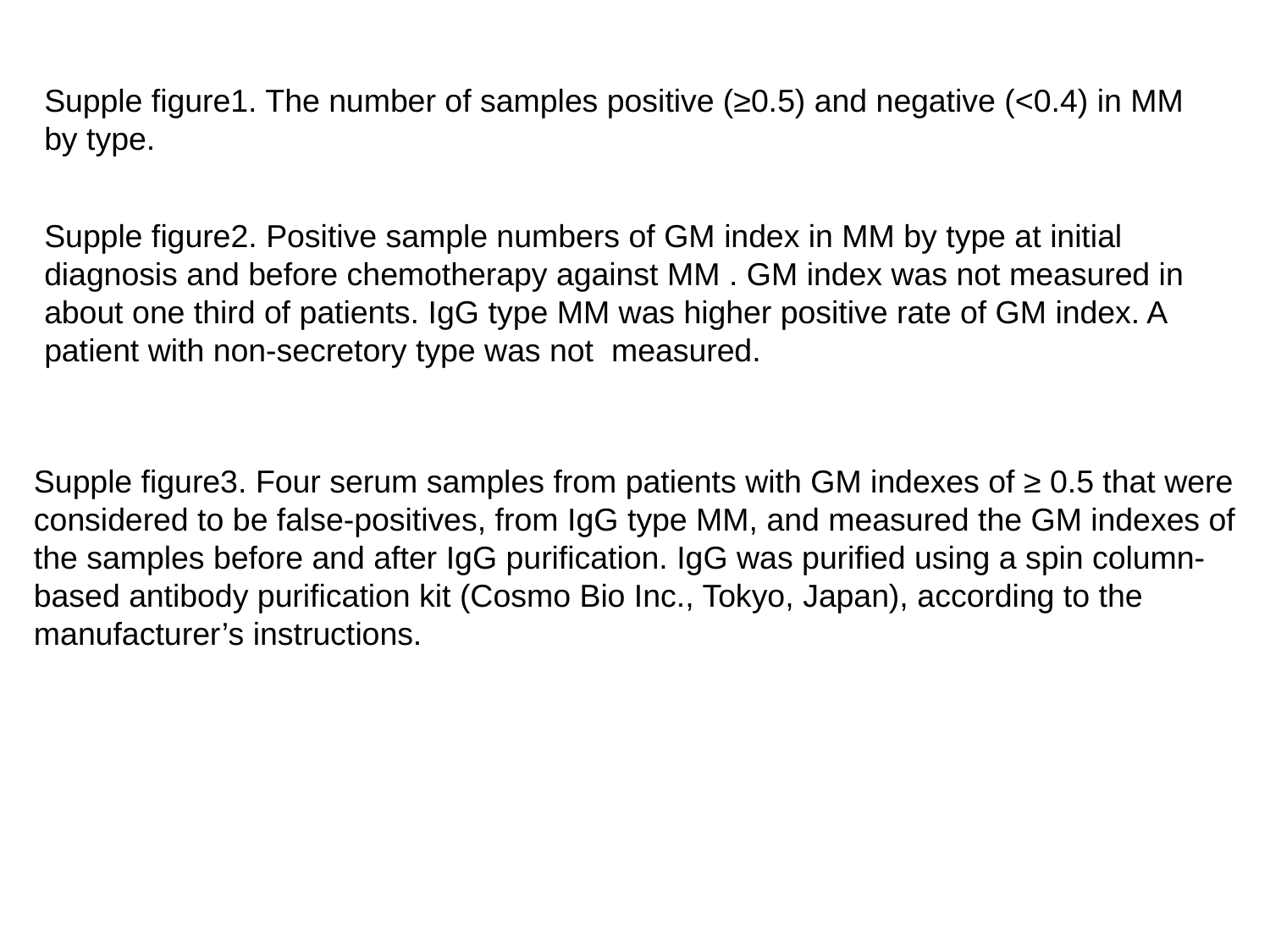

Supple figure1. The number of samples positive (≥0.5) and negative (<0.4) in MM by type.
Supple figure2. Positive sample numbers of GM index in MM by type at initial diagnosis and before chemotherapy against MM . GM index was not measured in about one third of patients. IgG type MM was higher positive rate of GM index. A patient with non-secretory type was not measured.
Supple figure3. Four serum samples from patients with GM indexes of ≥ 0.5 that were considered to be false-positives, from IgG type MM, and measured the GM indexes of the samples before and after IgG purification. IgG was purified using a spin column-based antibody purification kit (Cosmo Bio Inc., Tokyo, Japan), according to the manufacturer’s instructions.

## Slide 5
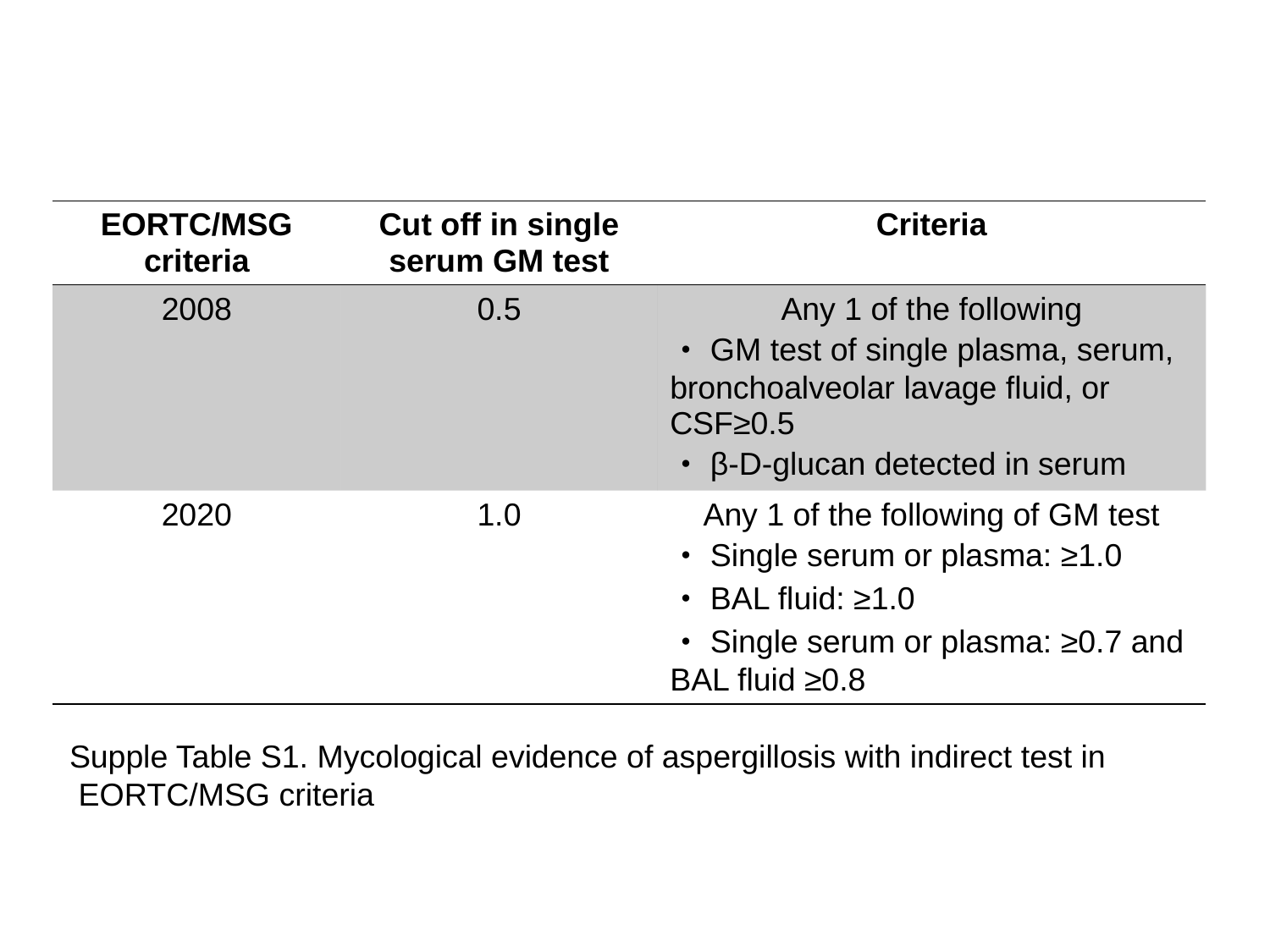

| EORTC/MSG criteria | Cut off in single serum GM test | Criteria |
| --- | --- | --- |
| 2008 | 0.5 | Any 1 of the following ・GM test of single plasma, serum, bronchoalveolar lavage fluid, or CSF≥0.5 ・β-D-glucan detected in serum |
| 2020 | 1.0 | Any 1 of the following of GM test ・Single serum or plasma: ≥1.0 ・BAL fluid: ≥1.0 ・Single serum or plasma: ≥0.7 and BAL fluid ≥0.8 |
Supple Table S1. Mycological evidence of aspergillosis with indirect test in
 EORTC/MSG criteria
